# Extra Virgin Olive Oil Phenolic Compounds: Modulating Mitochondrial Function and Protecting Against Chronic Diseases—A Narrative Review

**DOI:** 10.3390/nu17091443

**Published:** 2025-04-25

**Authors:** María Ángeles Silva-Soto, Paloma Carrillo-Fernández, Estefanía T. Saez Lancellotti, Elena Medina-Jiménez, Juan Francisco Mogaburo Alba, Nerea Catena-Granados, María Dolores López-Carmona, Luis Miguel Pérez-Belmonte, Nuria Prieto Lain, Ana Isabel Gómez Hernández, Ricardo Gómez-Huelgas, María-Rosa Bernal-López

**Affiliations:** 1 Internal Medicine Department, Regional University Hospital of Málaga, Instituto de Investigación Biomédica de Málaga (IBIMA-Plataforma Bionand), University of Málaga, Avda. Hospital Civil s/n, 29009 Málaga, Spain; angeles.silva@ibima.eu (M.Á.S.-S.); eslancellotti@gmail.com (E.T.S.L.); elena.mj2000@gmail.com (E.M.-J.); juframoal98@gmail.com (J.F.M.A.); ncgpsicologia@gmail.com (N.C.-G.); mdlcorreo@gmail.com (M.D.L.-C.); luismiguelpb@hotmail.com (L.M.P.-B.); prietolain@hotmail.com (N.P.L.); anabelgh96@hotmail.com (A.I.G.H.); rgh@uma.es (R.G.-H.); 2 UCM Digestive Diseases, Virgen del Rocío University Hospital, Instituto de Biomedicina de Sevilla (HUVR/CSIC/US), Department of Medicine, University of Seville, 41004 Seville, Spain; 3 Ciber Fisiopatología de la Obesidad y la Nutrición, Instituto de Salud Carlos III, 28029 Madrid, Spain

**Keywords:** Mediterranean diet (MedDiet), extra virgin olive oil (EVOO), polyphenols, mitochondria, aging

## Abstract

Background: Extra virgin olive oil (EVOO), an essential element of the Mediterranean diet (MedDiet), has demonstrated considerable potential in improving mitochondrial health and protecting against chronic diseases. This narrative review aims to explore how the main phenolic compounds found in EVOO—*hydroxytyrosol*, *oleuropein*, and *oleocanthal*—contribute to mitochondrial health by reducing oxidative stress and inflammation. Methods: A search for scientific evidence was carried out between October 2024 and March 2025 in different bibliographic databases such as PubMed, Web of Science, Embase, SciSpace, and ResearchRabbit databases. The search strategy included combinations of terms such as “extra virgin olive oil”, “EVOO polyphenols”, “mitochondrial function”, “oxidative stress”, “inflammation”, “mitophagy”, and “chronic diseases”. Preclinical, clinical, and mechanistic studies were included, giving priority to peer-reviewed publications. Results: This narrative review shows how some bioactive components of EVOO activate cellular pathways, such as mTOR, AMPK and sirtuins, which promote mitochondrial biogenesis, improve the efficiency of the electron transport chain, and protect mitochondrial DNA integrity. Furthermore, EVOO improves mitochondrial membrane fluidity and integrity, ensuring its functionality and efficiency. On the other hand, nutrition literacy, an important component of health, is a critical determinant of people’s eating behaviors. Conclusions: Although recent scientific evidence supports the metabolic benefits of EVOO components on mitochondrial metabolism and function, further nutritional intervention studies with these components are recommended to confirm their clinical relevance as a dietary tool aimed at preventing and/or delaying age-related metabolic diseases.

## 1. Introduction

The Mediterranean diet (MedDiet) has emerged as one of the principal dietary models with profound implications for public health. Adherence to the MedDiet is inversely associated with obesity, leading to greater weight loss and a reduced risk of chronic conditions such as cardiovascular disease (CVD) and certain cancers [1]. The MedDiet reflects a traditional dietary pattern shaped by centuries of local food practices in the Mediterranean region. This dietary pattern, which is characterized by the high consumption of fruits, vegetables, whole grains, and healthy fats, has been shown to improve overall health status and the quality of aging [2].

One of the defining characteristics of the Mediterranean diet across all regions is the abundant use of extra virgin olive oil (EVOO) as the primary added fat [3]. While the MedDiet is widely recognized for its role in preventing diverse diseases and is endorsed by global dietary guidelines, these recommendations still lack emphasis on distinguishing healthy fats, such as EVOO, from other fats and oils [3]. In addition, it has prebiotic properties, promoting beneficial gut bacteria and short-chain fatty acid production, which may contribute to overall health [4]. EVOO is rich in monounsaturated fatty acids (MUFAs) and phenolic compounds that have antioxidant and anti-inflammatory properties [5]. The regular consumption of EVOO is linked to a reduced risk of various chronic conditions, including CVD and cerebrovascular diseases, type 2 diabetes mellitus (T2DM), metabolic syndrome (MetS), cognitive decline, and certain cancers such as breast and colorectal [3].

EVOO also appears to lower the risk of obesity, prevent weight gain, and improve overall mortality [3]. EVOO supplementation has shown potential in mitigating cognitive impairment, particularly in neurodegenerative diseases (ND) such as Alzheimer’s disease (AD) [6].

Mitochondrial function is essential for adenosine triphosphate (ATP) production [7]. The process of β-oxidation, in which fatty acids are metabolized in the mitochondria, is crucial for maintaining energy balance and cell survival [8].

A decline in mitochondrial oxidative phosphorylation activity can lead to reduced ATP production and increased oxygen consumption, which adversely affects energy metabolism and contributes to metabolic disorders [9]. Mitochondrial dysfunction is a critical factor in conditions such as metabolic dysfunction-associated steatotic liver disease (MASLD), MetS and T2DM, contributing to oxidative stress (OS) and impaired energy metabolism [10]. Indeed, the integrity of mitochondrial function is vital for sustaining energy homeostasis, and its impairment can have profound implications [11].

The consumption of EVOO has been shown to positively influence mitochondrial function through various mechanisms, such as enhancing mitochondrial biogenesis, improving enzyme activity, and reducing OS [12]. This narrative review aimed to study the key evidence on the effects of EVOO and its bioactive components on mitochondrial function and dynamics and how this beneficial effect can help prevent diseases in which mitochondrial dysfunction is involved.

## 2. Methods

This narrative review aims to explore how the main phenolic compounds found in EVOO—*hydroxytyrosol* (HT), *oleuropein* (OLEU), and *oleocanthal* (OLEO)—contribute to mitochondrial health by reducing OS and inflammation and how this effect may influence diseases related to mitochondrial dysfunction. To gather the most relevant and updated evidence, we carried out literature searches between October 2024 and March 2025 using the PubMed, Web of Science, Embase, SciSpace, and ResearchRabbit databases. The search strategy included combinations of terms such as “extra virgin olive oil”, “EVOO polyphenols”, “mitochondrial function”, “oxidative stress”, “inflammation”, “mitophagy”, and “aging”. We focused on preclinical, clinical, and mechanistic studies, giving priority to peer-reviewed publications in English. We also manually reviewed the reference lists of selected papers to identify additional relevant sources.

**Figure 1:**
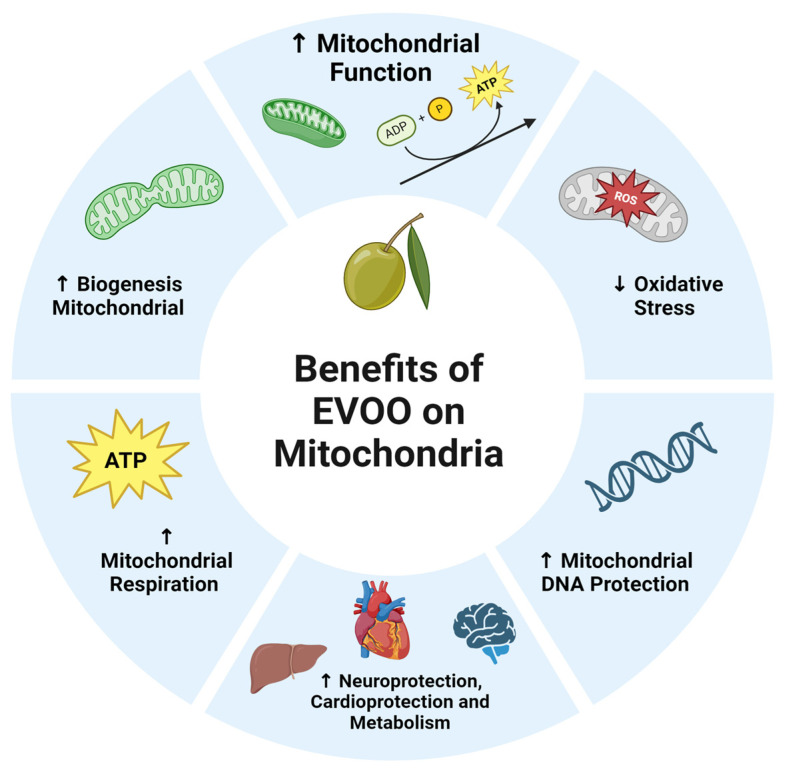
Benefits of EVOO on mitochondria. This figure was created using Biorender Program. It show how affect EVOO in mitochondrial metabolism, favoring the mitochondrial function as respiratory chain, mitochondrial biogenesis, DNA synthesis (up arrows) and decreasing (down arrows) oxidative stress. EVOO generates protective benefits at the brain, cardiovascular and metabolic levels.

Figure 1 was created using the Biorender Program version 04.

## 3. EVOO in the MedDiet

Adherence to the MedDiet is associated with numerous health benefits, including greater longevity and reduced risk of chronic diseases such as T2DM, MetS, obesity, CVD, and cancer [13]. Moreover, the MedDiet has been linked to favorable modifications in the gut microbiota composition. It promotes the growth of beneficial bacteria and reduces the proliferation of harmful species, which has a positive impact on inflammatory and oxidative status, metabolic health, and propensity for malignancy [14].

EVOO, the primary lipid source in the MedDiet, is rich in MUFAs and bioactive compounds such as phenols, triterpenes, and polyphenols, which are renowned for their antioxidant and protective properties [15,16]. These properties are mainly attributed to specific phenolic compounds, notably HT, OLEU, and *tyrosol* (Tyr), along with their derivatives, such as OLEO and *oleacein* (OLEA) [17,18].

## 4. EVOO Phenolic Compounds and Their Bioavailability

EVOO, derived from olives (*Olea europaea* L.), primarily consists of a saponifiable fraction (97–98%) and a non-saponifiable fraction (2%) that contains various bioactive elements [19]. The key component is triglycerides, with oleic acid as the dominant MUFA, while its smaller fraction contains over 200 compounds, including tocopherols, polyphenols (Tyr, HT, OLEU), sterols, phospholipids, waxes, squalene, and hydrocarbons [20]. Concentrations vary depending on the type of oil and period [21].

Simple phenols such as HT and Tyr; polyphenols such as flavonoids, *apigenin*, and *luteolin*; lignans such as *pinoresinol* and *1-acetoxypinoresinol*; and secoiridoids including OLEU, *glycosylated OLEU*, *demethyloleuropein*, *elenolic acid*, *ligstroside*, their aglycones, an isomer of the OLEU aglycon, and OLEO can be found in EVOO [22].

Phenolic compounds from olive oil (OO) are bioavailable in humans in a concentration-dependent manner [23]. The presence of an oil matrix can affect the bioaccessibility of polyphenols, leading to a significant increase in the bioaccessibility of certain polyphenols when consumed together with oils [24]. HT and Tyr are more efficiently absorbed than other phenolic compounds, and their levels increase significantly in the small intestine after gastric digestion [21].

The European Food Safety Authority (EFSA) has recognized HT and OLEU, along with related compounds, as the agents responsible for the beneficial effects of EVOO [25]. Studies suggest that the effects of OLEU are largely due to its conversion into HT, with minimal plasma levels of OLEU and increased HT levels after OLEU consumption [26].

## 5. Effects of Olive Oil Phenolic Compounds on Mitochondrial Function

EVOO positively influences mitochondrial biogenesis and antioxidant capacity. Polyphenols such as OLEU and HT enhance mitochondrial function by reducing OS; improving energy efficiency; and regulating genes linked to OS, inflammation, and lipid metabolism [27,28] (Figure 1).

### 5.1. Improvements in Mitochondrial Biogenesis

EVOO promotes mitochondrial biogenesis—the formation of new mitochondria—which is a key process for cellular homeostasis, energy production, and stress response. It is driven by factors including energy demand, stressors, and metabolic changes, which influence health and aging [29,30]. HT may boost mitochondrial biogenesis by triggering the AMP-activated protein kinase (AMPK) pathway, which upregulates key genes like the peroxisome proliferator-activated receptor gamma coactivator 1-alpha (PGC-1α), nuclear respiratory factor 1 (NRF-1), and mitochondrial transcription factor A (TFAM) that are essential for mitochondrial growth and replication [31]. In vitro studies have found that HT stimulates mitochondrial biogenesis in adipocytes, enhancing mitochondrial DNA (mtDNA) levels and efficiency by activating PGC1α, which promotes mitochondrial complex synthesis and oxygen consumption [32].

The effects of ten different purified phenolic secoiridoids on mitochondrial performance were investigated in a mouse model of early AD. *Ligstroside* was found to be the most potent mitochondrial bioenergetic enhancer. This compound increased ATP levels in the brains of mice. It also improved the mRNA expression of genes involved in mitochondrial biogenesis [33]. Moreover, in high-fat diet (HFD) fish models, HT reduced liver fat accumulation, lowered reactive oxygen species (ROS) levels, increased AMPK expression, upregulated autophagy genes, and restored mtDNA copy number [34].

### 5.2. Oxidative Stress Reduction

Cellular redox homeostasis, or the balance of oxidants and antioxidants, is crucial for proper cell function. EVOO polyphenols support this balance by reducing ROS, increasing antioxidant levels, and activating signaling pathways that protect mitochondria and enhance the stress response [35]. Nuclear factor kappa-light-chain-enhancer of activated B cells (NF-κB) and nuclear factor erythroid 2-related factor 2 (Nrf-2) are key transcription factors that regulate responses to OS and inflammation. EVOO polyphenols, including HT, Tyr, and OLEU, can influence these pathways. Nrf-2 activation promotes cytoprotective gene transcription that strengthens cellular defenses against oxidative damage and inflammation [36]. EVOO polyphenols increase Nrf-2 activation, strengthening cellular protective mechanisms while suppressing NF-κB activity, a major driver of inflammation [37]. Specifically, HT and Tyr effectively modulate NF-κB signaling by downregulating pro-inflammatory cytokine expression and directly inhibiting NF-κB activation. This dual action curtails inflammatory cascades, providing protection against OS. Additionally, HT and Tyr regulate mitochondrial function, reducing ROS production at its source, which further minimizes oxidative damage [38].

OLEU has been shown to increase intracellular calcium ion (Ca^2+^) levels, activating AMPK and PGC-1α, which regulate cellular energy homeostasis and mitochondrial biogenesis. Additionally, OLEU interacts with the SIRT1 pathway, reducing mitochondrial ROS production and fostering stress resistance and cellular longevity through improved mitochondrial function and decreased OS [39].

### 5.3. Mitochondrial Respiration Improvement

Mitochondria are essential organelles known for their role in energy production via oxidative phosphorylation [40]. Mitochondrial respiratory enzyme function is vital for protecting against disease since it is essential for maintaining cellular energy levels [41].

EVOO enhances the functionality of the mitochondrial electron transport chain (ETC), increasing ATP production. Studies highlight its benefits in aging, with experiments in older rats showing restored mitochondrial enzyme levels after six weeks of EVOO consumption. This intervention improved overall mitochondrial function and preserved ETC enzyme activities, particularly NADH-CoQ oxidoreductase (complex I) and cytochrome c oxidase (complex IV), protecting against age-related mitochondrial decline [42]. HT improves mitochondrial complex activities [32], increasing oxygen consumption in adipocytes and enhancing cellular respiration and metabolic function. Secoiridoids, particularly OLEA, OLEU, OLEO, and *ligstroside*, significantly elevate ATP levels and enhance the capacity of respiratory chain complexes, further supporting mitochondrial efficiency [33].

### 5.4. Mitochondrial DNA Protection

EVOO phenolic compounds influence mtDNA through OS modulation and methylation patterns [43]. Likewise, improves mitochondrial function by regulating pathways such as the AMPKα/SIRT1/mTOR pathways, which are key for autophagy and metabolic homeostasis [12]. It is also associated with increased mtDNA methylation, suggesting an epigenetic role. Additionally, EVOO improves mitochondrial membrane fluidity and integrity, prevents DNA damage, and supports DNA repair mechanisms. Secoiridoids in EVOO strengthen defense systems, preserving genomic integrity and fostering cellular repair. Additionally, dietary OO has been linked to increased mtDNA [44]. The combination of EVOO and exercise increased the levels of muscle cytochrome c and PGC-1 α, increased autophagy markers, and decreased lipid peroxidation in muscle [45].

The aging process compromises physiological functions and entails inflammation and diminished autophagy. HT and OLEU have been shown to be efficacious in enhancing mitophagy, improving mitochondrial functionality, and boosting cognitive performance in AD animal models [46].

The impact of highly purified olive secoiridoids (POS) on ATP levels was assessed in a well-established murine model of age-related brain changes. Mice were administered either POS with OLEU, HT, and *oleurosid* or a control diet that did not contain POS for six months. Older mice had reduced ATP levels and mRNA expression, suggesting mitochondrial impairment. Additionally, the expression of genes Sirt1, CREB, Gap43, and GPx-1 was significantly diminished in aged brain tissues. POS supplementation led to a restoration of ATP levels in aged mice. Furthermore, mice that received POS showed enhanced spatial working memory. These improvements are linked to elevated ATP levels in the brain [47]. HT has also been demonstrated to have a protective effect in specific forms of cancer and to modulate the pathways that control energy metabolism [48].

The impact of oleate and palmitate on hepatic cells has been previously studied and found to have dose- and time-dependent influences on mitochondrial dynamics. Mitofusin 2 (MFN2) plays a crucial role in mitochondrial fusion/fission, metabolism, and interactions with organelles such as the endoplasmic reticulum [49]. Oleate enhances MFN2 levels and cell viability more effectively than palmitate. Palmitate induces more apoptosis than oleate, which may protect against mitochondrial dysfunction [50].

Similarly, OLEU activates antioxidant mechanisms via the Nrf-2 pathway, essential for gene activation of antioxidant enzymes like superoxide dismutase (SOD) and catalase. Furthermore, OLEU decreases DNA denaturation, mitochondrial ROS, and superoxide anion levels [51].

## 6. Mechanisms of Action

### 6.1. Antioxidant Effects

Mitochondria-targeted antioxidants, such as those derived from EVOO, can effectively cross the mitochondrial membrane, directly addressing ROS at their source [52].

OO phenolic compounds exhibit specific antioxidant mechanisms in mitochondria, mitigating the oxidative damage associated with aging and various diseases [12]. Additionally, oleic acid activates glutathione peroxidase via epidermal growth factor receptor (EGFR) signaling, which further decreases ROS levels in the mitochondria, enhancing cellular antioxidant defenses [53].

The ability of the phenolic compounds in OO to participate in proton-coupled electron transfer (PCET) reactions is considered the reason for their antioxidant properties [54]. This sophisticated mechanism allows these phenols to effectively neutralize ROS.

EVOO phenolic compounds like HT and OLEU counteract OS and neuroinflammation. This has been shown in epidemiological studies that link high OO consumption to improved cognitive performance in older adults [55]. Additionally, OO’s antioxidant properties are enhanced by the polarity of its compounds, which affects their dispersibility and effectiveness in protecting against oxidation [56].

OLEO has also been shown to mitigate OS in neuronal cells by increasing cell viability and reducing ROS production, suggesting a protective role against mitochondrial dysfunction [57]. In addition, the fatty acids in EVOO activate glutathione peroxidase through EGFR signaling, thus enhancing antioxidant defenses against mitochondrial OS [53].

### 6.2. Anti-Inflammatory Effects

The EVOO polyphenols can influence the modulation of the inflammatory processes at the mitochondrial level via multiple mechanisms. These polyphenols activate nutrient-sensing stress-response pathways that influence immune responses and metabolic pathways, promoting an immunophenotype with less inflammation [58].

Reduced mitochondrial function is associated with chronic inflammation, obesity, and MetS. This impairment could affect the production and efficiency of energy in the mitochondria, a phenomenon observed in many cardiometabolic conditions linked to obesity [36]. Polyphenol-rich EVOO has been shown to improve mitochondrial activity and fatty acid oxidation in HFD models, alleviating liver inflammation and insulin resistance, as well as improving glucose regulation and insulin sensitivity [59]. Furthermore, EVOO polyphenols trigger the AMPK and Nrf2 pathways, exhibiting anti-inflammatory properties. Specifically, *OLEU aglycone* triggers Nrf2-controlled vitagenes, protecting against ND, such as AD and Parkinson’s disease [60].

Torres-Sánchez et al. proposed a relationship between dietary lipids, membrane stability, and mitochondrial efficacy in patients with multiple sclerosis (MS), a disease characterized by central nervous system inflammation together with increased OS and mitochondrial dysfunction. Patients received fish or OO supplements after six or nine months; both oils improved mitochondrial membrane fluidity [61].

In addition, OLEU influenced the cytokine expression and significantly reduced the levels of M1-associated pro-inflammatory cytokines such as IL-12, IFN-γ, and TNF-α, suggesting a shift towards an anti-inflammatory response. In contrast, OLEU increases the expression and production of M2-associated anti-inflammatory cytokines, such as IL-10 and TGF-β, thus promoting a favorable anti-inflammatory environment [62].

## 7. Mitochondrial Impact of Olive Oil in Disease

Mitochondria not only generate ATP but are also involved in various cellular processes, such as calcium levels, cell death pathways, and the transmission of ROS signals [63]. They intervene in cellular signaling by interacting intracellularly and extracellularly in several ways [64]. Mitochondria also release signaling molecules, known as mitokines, in response to stress. Mitokines can affect distant tissues and organs, thereby contributing to the beneficial effects of exercise on systemic health [65].

Many factors contribute to developing mitochondrial dysfunction, including changes in DNA sequence, aging, infections, and a sedentary lifestyle [66]. Mitochondrial dysfunction leads to increased ROS levels, which can disrupt cellular metabolism, redox balance, and apoptotic pathways. This may contribute to the development of conditions such as cancer, T2DM, infections, obesity, and ND [67,68].

Fission and fusion processes maintain mitochondrial function. Mitophagy eliminates damaged components during fission, whereas fusion connects healthy segments to facilitate repair. Aging frequently causes a decline in mitochondrial dynamics, characterized by more fission and less fusion [69].

At physiological concentrations, ROS serve as important signaling molecules that maintain cellular stability. Mitochondria have a structured antioxidant system that efficiently scavenges most of the ROS generated within this organelle [31]. In contrast, excessive ROS production beyond antioxidant capacity can lead to cellular dysfunction and damage to mitochondrial DNA, proteins, and lipids. This can disrupt mitochondrial function and cellular balance [66].

Limited clinical evidence available at present suggests that OO consumption positively impacts mitochondrial function in patients with chronic diseases, particularly through its phenolic compounds and nitro-fatty acids [12].

### 7.1. Cardiovascular Health

Aging is one of the major risk factors for CVD, which has an oxidative pathophysiological component [70]. Mitochondria are highly efficient in meeting the high energy demands of heart muscle contractions and occupy about a third of the area of adult heart muscle cells. In this context, mitochondrial dysfunction refers to a state in which the mitochondria are unable to meet the cell’s demand for ATP, and there is increased ROS formation [71]. This dysfunction can occur as a result of mtDNA and/or nDNA mutations but also in response to various diseases or environmental stress, leading to the development of CVD [71].

Mitochondrial dysfunction has been correlated with increased ROS production in CVD, resulting in a diminished mitochondrial membrane potential. This, in turn, activates a range of signaling proteins and initiates the process of apoptosis [72]. Mitochondria are highly responsive to variations in nutrient and oxygen availability, adjusting their metabolic processes in response to shifts in both the intracellular and extracellular environments [73]. In CVD, this adaptive mechanism is impaired, resulting in a gradual deterioration of mitochondrial function linked to ETC dysfunction, ATP production issues, heightened OS, and compromise of the structural integrity [74].

The cardioprotective potential of OLEU, HT, and OLEO, particularly in the context of acute myocardial infarction and MetS, has been studied, and significant benefits have been found. For instance, OLEU has shown the capability to reduce myocardial ischemia/reperfusion damage by inhibiting OS via the TLR4/MAPK signaling pathway [75]. The combination of OLEU, HT, and OLEO demonstrated protective benefits, such as diminished infarct size and better blood sugar level regulation, due to increased antioxidant properties and the inhibition of apoptosis [76].

Consumption of EVOO may positively impact mitochondrial function and cardiovascular health in patients with CVD, improve endothelial function, reduce OS, and enhance myocardial performance, as evidenced by significant improvements in markers such as flow-mediated dilation and coronary flow reserve in patients with stable coronary artery disease after supplementation with an enriched OO extract [77]. Additionally, high OO consumption correlates with a lower risk of coronary heart disease and overall cardiovascular morbidity and mortality, reinforcing its cardioprotective properties [78]. Lastly, OO has been shown to mitigate mitochondrial OS, enhancing the efficiency of the mitochondrial ETC [79].

### 7.2. Neuroprotection

Mitochondrial energy deficiency is one of the hallmarks of ND [80]. EVOO polyphenols have been shown to mitigate OS and neuroinflammation, both of which are critical factors in NDs like AD and Parkinson’s disease [81].

Higher consumption of EVOO has been correlated with improved cognitive performance and a reduced risk of ND [55]. Clinical trials have demonstrated that EVOO enhances blood-brain barrier integrity and cognitive function in individuals with mild cognitive impairment, suggesting a potential to delay cognitive decline [82].

The MedDiet, rich in OO, has shown benefits in ND by improving mitochondrial function and antioxidant levels, which are essential for handling OS related to diseases like amyotrophic lateral sclerosis (ALS) [83]. HT has also been demonstrated to improve mitochondrial energetics by promoting mitochondrial biogenesis and reducing ROS production [84]. In addition, HT has been shown to reduce mitochondrial carbonyl proteins, increase SOD, and decrease the levels of inflammatory markers in the brain, thereby regulating mitochondrial OS, neuroinflammation, and apoptosis [85].

### 7.3. Metabolic Disorders

Metabolic diseases—including obesity, hypertension, dyslipidemia, T2DM or MASLD—are a group of disorders that affect the body’s ability to process and utilize nutrients. Some studies have highlighted the contribution of EVOO bioactive compounds, such as Tyr, OLEO, and HT, in mitigating these conditions through mitochondrial function preservation and promotion [31].

Diets rich in OO are known to improve mitochondrial function, gut microbiota diversity, insulin sensitivity, and reduced inflammation in rodent models [59]. Notably, OLEU and HT enhance lipid and glucose metabolism by activating the AMPKα/SIRT1/mTOR pathway, promoting fatty acid β-oxidation, autophagy, and positively regulating key genes such as PPARα and CPT1, facilitating GLUT4 translocation in muscle cells [12,32].

EVOO-derived nitro-fatty acids have been shown to protect against mitochondrial oxidative damage, improving mitochondrial respiratory function and reducing fat accumulation in the liver [86]. Additionally, the administration of HT in both in vitro and in vivo aquatic models significantly augments mitochondrial functionality in MASLD, decreasing lipid deposition, OS, and mitochondrial impairment through the facilitation of mitophagy mediated by the AMPK/PINK1 signaling pathway [87]. The incorporation of EVOO into the diet has been demonstrated to rehabilitate mitochondrial dynamics by equilibrating the processes of fusion and fission [45].

Furthermore, OO has been shown to increase mitochondrial content and fatty acid oxidation in liver and heart tissues in mice [88]. Dietary EVOO supplementation has been reported to reduce SREBP-1c expression, hepatic triglyceride content and lipogenesis in corpulent JCR:LA-cp rats [89]. HT inhibits the SREBP-1c/FAS pathway, leading to a decrease of HFD-induced lipid deposits, ameliorating OS and promoting antioxidant activity in both liver and skeletal muscle in C57BL/6J mice. Remarkably, HT has also been shown to decrease fasting glucose in the same way as metformin [90].

However, other studies suggest that OO does not alter metabolic parameters, although when combined with conjugated linoleic acid, it demonstrates synergistic metabolic benefits, including white adipose tissue reduction, energy expenditure increase, or liver mitochondrial uncoupling protein-2 (UCP-2) expression increase [91] (Table 1).

## 8. Nutrition Literacy and Eating Behaviors

Nutrition literacy, which is one of the important components of health literacy, has become a critical determinant in shaping people’s eating behaviors. This includes basic nutritional information and understanding, as well as interpreting and having the ability to make healthy decisions on nutrition-related issues, particularly with regard to functional foods such as EVOO [92,93].

Positive correlations have been demonstrated between higher levels of nutritional literacy and better adherence to the MedDiet in various populations [94,95,96]. Higher nutrition literacy has been specifically associated with increased consumption of key MedDiet components, including EVOO, fruits, vegetables, legumes, and nuts, reflecting a better appreciation of their nutritional value and health benefits [94,95].

Individuals with better nutrition literacy demonstrate an enhanced ability to interpret food labels, understand portion sizes, and apply nutritional knowledge to meal planning and preparation, leading to better dietary choices [94]. Furthermore, nutrition literacy has been linked to reduced consumption of ultra-processed foods, added sugars, and unhealthy fats, supporting better overall diet quality [96,97]. Interestingly, a study of university students found that while 84.1% demonstrated adequate nutrition literacy, their adherence to the MedDiet remained low [98]. This discrepancy highlights the complex relationship between nutritional knowledge and eating behavior, where adequate information may not be sufficient to make optimal dietary choices [98].

Sociodemographic factors impact nutrition literacy, with education being a key predictor. Specifically, higher education levels correlate with better nutrition literacy [93,99,100]. The relationship between nutritional literacy and eating behaviors appears consistent across different population groups, although specific skills may vary by age, cultural context, and socioeconomic status [99]. Women tend to have higher nutritional literacy than men [96,100]. Age affects nutritional literacy in complex ways, and some studies show a decline in certain aspects among older adults, especially in rural areas [99]. Health professionals tend to have higher nutrition literacy than other occupational groups [100].

Considering the strong relationship between nutritional literacy and adherence to the MedDiet, educational strategies that promote understanding of MedDiet patterns and the health benefits of key components such as EVOO will be effective in improving diet quality [92,96].

Targeted interventions with age-appropriate content and methods emphasizing practical skills may be more effective than those focused only on knowledge transfer [101]. Technology-driven nutrition education, through apps, websites, and social media, expands reach and delivers personalized, practical guidance [102].

## 9. Discussion

EVOO, as an essential component of the MedDiet, has been shown to have considerable potential in improving mitochondrial health [12]. Its main phenolic compounds, such as HT, OLEU, and OLEO, help preserve mitochondrial functionality by reducing OS and inflammation [27,28,29,30]. These bioactive components activate key cellular pathways, such as mTOR, AMPK, and sirtuins, which are known to promote mitochondrial biogenesis, improve the efficiency of the ETC, and protect mtDNA integrity [12]. Furthermore, EVOO improves mitochondrial membrane fluidity and integrity, ensuring its functionality and efficiency [31,32,33,34,35,36,37,38,39,40,41,42].

The MedDiet is characterized by high consumption of fruits, vegetables, whole grains, and healthy fats [2] and is associated with several health benefits, including increased longevity and lower risk of chronic diseases [11,12,13] such as T2DM, MetS, obesity, CVD, and cancers such as breast and colorectal cancer [3,13]. EVOO, the primary lipid source in the MedDiet [15], is rich in MUFAs and bioactive compounds such as phenols, triterpenes, and polyphenols, known for their antioxidant and protective properties [16]. These properties are mainly attributed to specific phenolic compounds, notably HT, OLEU, and Tyr, along with their derivatives, such as OLEO and OLEA [16]. The presence of an oily matrix can affect the bioaccessibility of polyphenols, leading to a significant increase in the bioaccessibility of certain polyphenols when consumed together with oils [24]. The EFSA has recognized HT and OLEU, along with related compounds, as the agents responsible for the beneficial effects of EVOO [25].

HT can stimulate mitochondrial biogenesis, a key process for cellular homeostasis and energy production, by activating the AMPK pathway, which upregulates key genes such as PGC-1α, NRF-1, and TFAM, which are essential for mitochondrial growth and replication [29,30,31].

EVOO polyphenols also support cellular redox homeostasis by reducing ROS, increasing antioxidant levels, and activating signaling pathways that protect mitochondria and enhance the stress response [35,36,37,38,39]. Furthermore, EVOO enhances the functionality of the mitochondrial ETC, increasing ATP production [40] and protecting against age-related mitochondrial decline [41].

EVOO phenolic compounds influence mtDNA through OS modulation and methylation patterns [43]. It also, EVOO improves mitochondrial membrane fluidity and integrity, prevents DNA damage, and supports DNA repair mechanisms [12].

These effects of EVOO phenolic components have implications for various diseases. In cardiovascular health, mitochondrial dysfunction promotes the development of CVD [70,71]. EVOO consumption may have a positive effect on patients with CVD, improving endothelial function, reducing OS, and enhancing myocardial performance [77]. In neuroprotection, mitochondrial energy deficiency is a hallmark of ND [80,81]. EVOO polyphenols can mitigate OS and neuroinflammation [80,81]. Higher EVOO consumption has been correlated with improved cognitive performance and lower risk of ND [55]. In metabolic disorders, including obesity, T2DM, or MASLD, EVOO phenols such as Tyr, OLEO, and HT may help mitigate these conditions by improving mitochondrial function [31].

Nutritional literacy has become an important determinant of people’s eating behaviors [92,93]. Positive correlations have been demonstrated between higher levels of nutrition literacy and better adherence to the MedDiet in several populations [94,95,96]. Nevertheless, the relationship between nutritional knowledge and eating behavior is complex, and adequate information may not be enough to make optimal dietary choices [94].

However, it is important to acknowledge that most of the current evidence comes from preclinical studies, and human data remains limited. While EVOO polyphenols are considered safe for dietary intake, recent research has raised some concerns regarding excessive or pharmacological doses. At high concentrations, polyphenols may exhibit prooxidant behavior, interfering with mitochondrial signaling pathways and redox homeostasis [103]. This knowledge reminds us that context matters and that the benefits of EVOO may depend on its consumption as part of a healthy and balanced dietary pattern.

## 10. Conclusions

EVOO is an essential component of the MedDiet. It benefits mitochondrial health through various mechanisms. This beneficial effect is mainly attributed to its MUFA content and its bioactive phenolic compounds, such as HT, OLEU, and OLEO. These components exhibit antioxidant, anti-inflammatory, and metabolic regulatory properties.

Research suggests that EVOO promotes mitochondrial biogenesis and energy production, improving mitochondrial respiratory efficiency and reducing OS. By activating key signaling pathways, such as the AMPKα/SIRT1/mTOR, EVOO promotes both autophagy and mitophagy, contributing to metabolic homeostasis and cell regeneration. These effects are particularly relevant in cardiovascular, neurodegenerative, and metabolic diseases in which mitochondrial dysfunction is involved. Additionally, EVOO has been shown to improve mitochondrial membrane fluidity and protect mitDNA, thus reinforcing genetic stability and repair mechanisms.

Altogether, these findings suggest that EVOO may be a valuable nutritional tool with potential benefits in the prevention of diseases associated with aging and mitochondrial dysfunction.

Nutrition literacy is a key factor in shaping dietary choices, including adherence to the MedDiet and informed consumption of EVOO, presenting challenges and opportunities for targeted interventions.

However, while preclinical studies are promising, further research in humans is needed to better understand and confirm its clinical benefits.

## 11. Future Research Directions

To better understand the long-term impact of EVOO on mitochondrial health and related diseases, well-designed longitudinal studies in humans are needed. It would be particularly relevant to investigate whether regular EVOO consumption can help prevent or slow the progression of age-related conditions where mitochondrial dysfunction plays a central role, such as sarcopenia.

Another key question is which doses of EVOO polyphenols—particularly *hydroxytyrosol* (HT) and *oleuropein* (OLEU)—are most effective in enhancing mitochondrial function. Comparative studies are also needed to determine whether these compounds provide unique advantages over other antioxidants and how their effects compare specifically to omega-3 fatty acids from fish oil in supporting mitochondrial health. It will be important to clarify whether their mechanisms of action are complementary or distinct.

Finally, future research should examine how integrating nutrition literacy into educational settings, clinical practice, and public health initiatives can improve adherence to dietary patterns that support mitochondrial function and promote healthy aging.

## Figures and Tables

**Table 1:** Effects of EVOO and Its Compounds on Mitochondrial Function.

Reference	Effects on Mitochondria	Study Type	Year	Intervention
[32]	↑ Mitochondrial biogenesis, fatty acid oxidation, complexes I-V activity, PPARGC1α expression	In vitro	2010	Hydroxytyrosol
[33]	↑ Brain ATP levels, ↓ Aβ 1-40 (AD model), ↑ mRNA mitochondrial biogenesis genes	In vitro and in vivo	2020	Oleocanthal and Ligstroside
[34]	↓ Hepatic fat deposition, ↓ ROS, ↑ p-AMPK, ↑ autophagy genes	In vivo	2020	Hydroxytyrosol
[39]	↑ AMPK, PGC-1α, intracellular Ca^2+^ concentration, ↓ mitochondrial ROS	In vitro	2022	Oleuropein
[42]	↑ Mitochondrial enzyme activity	In vivo	2015	EVOO
[45]	↑ Muscle cytochrome c, PGC-1α, mitochondrial fusion proteins, ↓ lipid peroxidation	In vivo	2022	EVOO + Training
[47]	↑ Brain ATP levels	In vivo	2018	Purified Olive Secoiridoids
[50]	↑ MFN2 (mitochondrial fusion)	In vitro	2021	Oleate
[59]	↓ Liver inflammation, ↑ fatty acid oxidation, glucose homeostasis, ↓ NAFLD progression	In vivo	2017	EVOO
[61]	↑ Mitochondrial membrane fluidity, ↓ ATP hydrolysis in RR-MS patients	Controlled Trial	2018	EVOO
[86]	↑ NO2-FA formation, mitochondrial function, respiratory indexes, complex activity (NAFLD model)	In vivo	2021	EVOO + Nitrite
[87]	↓ Fat accumulation, oxidative stress, ↑ PINK1-mediated mitophagy	In vivo	2022	Hydroxytyrosol

Abbreviations: ATP, adenosine triphosphate; EVOO, extra virgin olive oil; MFN2, mitofusin-2; NAFLD, non-alcoholic fatty liver disease; ROS, reactive oxygen species; RR-MS, relapsing-remitting multiple sclerosis. Up arrows indicate an increase and down arrows indicate a decrease of different metabolic processes.

## Abbreviations

The following abbreviations are used in this manuscript:

AD	Alzheimer’s disease
AMPK	AMP-activated protein kinase
ATP	Adenosine triphosphate
Ca^2+^	Ion de calcio intracelular
CVD	Cardiovascular disease
DRP1	Dynamin-related protein 1
EGFR	Epidermal growth factor receptor
EFSA	European Food Safety Authority
ETC	Electron transport chain
EVOO	Extra virgin olive oil
HFD	High-fat diet
HT	Hydroxytyrosol
MASLD	Metabolic dysfunction-associated steatotic liver disease
MedDiet	Mediterranean diet
MetS	Metabolic syndrome
MFN2	Mitofusin 2
mtDNA	Mitochondrial DNA
mTOR	Mammalian Target of Rapamycin
MUFAs	Monounsaturated fatty acids
mRNA	Messenger RNA
ND	Neurodegenerative diseases
NF-κB	Nuclear factor kappa-light-chain-enhancer of activated B cells
Nrf-2	Nuclear factor erythroid 2-related factor 2
NRF-1	Nuclear respiratory factor 1
OLEA	Oleacein
OLEO	Oleocanthal
OLEU	Oleuropein
OO	Olive oil
OS	Oxidative stress
PGC-1α	Peroxisome proliferator-activated receptor gamma coactivator 1-alpha
POS	Highly purified olive secoiridoids
ROS	Reactive oxygen species
SIRT1	Sirtuin 1
SOD	Superoxide dismutase
T2DM	Type 2 diabetes mellitus
TFAM	Mitochondrial transcription factor A
Tyr	Tyrosol
UCP-2	Uncoupling protein-2
